# Current perspectives on skeletal health and cancer progression across the disease continuum in breast cancer—the role of bisphosphonates

**DOI:** 10.3332/ecancer.2012.265

**Published:** 2012-08-23

**Authors:** R Aft

**Affiliations:** 1 Department of Surgery, Washington University School of Medicine, 660 South Euclid Avenue, St. Louis, MO 63110, USA

**Keywords:** *bisphosphonates*, *breast cancer*, *treatment*, *zoledronic acid*

## Abstract

Pre-clinical and clinical evidence suggest that bisphosphonates inhibit both bone resorption and cancer progression. New and updated analyses from several large, controlled studies in pre- and post-menopausal women with early stage breast cancer (BC) suggest that addition of bisphosphonates improves cancer-related outcomes, particularly in patients with a ‘low-estrogen environment’. Further, preliminary clinical data suggest that bisphosphonate therapy may reduce circulating tumour cell numbers (a negative prognostic indicator of disease-free and overall survival) in patients with advanced/metastatic disease. These new findings warrant reconsideration of the therapeutic role of bisphosphonates in BC.

## Introduction

Bisphosphonates are the most common pharmaceutical intervention for prevention of skeletal-related events (SREs) in patients with malignant skeletal involvement. Data from several studies suggest that in addition to inhibiting bone resorption, bisphosphonates may limit cancer progression through their effects within the bone (e.g. cancer cell–bone interactions) or their effects on extraskeletal processes such as host antitumour immunity, angiogenesis, and circulating tumour cells (CTCs). The potential of simultaneously limiting bone resorption and tumourigenesis by bisphosphonates is therapeutically relevant, and accordingly, several large, clinical programs have evaluated the anticancer benefits of adding bisphosphonates to standard-of-care in the adjuvant setting in earlier stage breast cancer (BC) and in patients with advanced/metastatic BC. These studies shed light on the anticancer benefits of bisphosphonate therapy and the subsets of patients who may benefit from such therapy. Recently, several large, controlled studies in patients with earlier stage disease reported data suggestive of potential anticancer benefit of zoledronic acid (ZOL) in particular patient subsets. In addition, smaller studies suggest that ZOL may have similar anticancer benefits in patients with advanced/metastatic disease. These data warrant reconsideration of clinical practice in early BC and further clinical exploration of the therapeutic role of bisphosphonates in advanced BC.

## Early disease

Several trials have demonstrated the anticancer benefit of adding bisphosphonates to standard adjuvant treatment in patients with early stage disease (Table 1) [1–5]. For example, in the ABCSG-12 study in pre-menopausal patients with early BC undergoing complete estrogen blockade (a patient population highly susceptible to bone loss), 3 years’ ZOL treatment (4 mg every 6 months) significantly improved disease-free survival (DFS) at the 48-month follow-up (hazard ratio [HR] = 0.74; log-rank *P* = 0.01); moreover, these benefits were maintained at 62 (HR = 0.68; log-rank *P* = 0.008), 76 (HR = 0.73; log-rank *P* = 0.02), and 84 months (HR = 0.71; log-rank *P* = 0.011), demonstrating potential carryover anticancer benefit persisting for 4–5 years after treatment cessation [2]. Moreover, at the 84-month follow-up, analysis of overall survival (OS) showed that ZOL decreased risk of death versus placebo (HR = 0.61; *P* = 0.033) and that this benefit appeared to be driven by the subset of patients >40 years of age (HR = 0.57; *P* = 0.042).

In the NSABP-B34 study evaluating the benefit of adding clodronate to adjuvant therapy in patients with earlier stage BC, clodronate treatment was associated with significant improvement in non-bone metastasis-free survival (HR = 0.743; *P* = 0.046), although there was no observable difference between treatment arms in the DFS primary endpoint (HR = 0.91; *P* = 0.27) [3]. More importantly, however, anticancer benefits were more marked in the subset of patients >50 years of age (recurrence-free interval: HR = 0.76, *P* = 0.05; bone metastases-free interval [BMFI]: HR = 0.61, *P* = 0.024; non-BMFI: HR = 0.63, *P* = 0.015; OS: HR = 0.80, *P* = 0.1) [3].

Consistent with these data, Coleman *et al* reported that although adjuvant ZOL had no effect on survival in the overall study population of BC patients in the AZURE trial, ZOL was associated with survival benefit in the subset of patients with a low-estrogen environment (>5 years post-menopause at study entry) [6]. Similarly, in an exploratory analysis of data from the ZO-FAST study, initiation of ZOL concurrent with standard adjuvant therapy significantly improved survival (HR = 0.50, *P* = 0.0224) in early stage BC patients who were >5 years post-menopause or >60 years of age [7]. In the GAIN study, a similar trend toward improved survival was noted in ibandronate-treated patients ≥60 years of age (HR = 0.746, *P* = 0.172) [4].

More recently, the novel receptor activator of nuclear factor kappaB ligand (RANKL)-directed antibody denosumab was shown to significantly increase bone mineral density (BMD) over 24 months at trabecular and cortical bone in women with non-metastatic BC and low-bone mass receiving adjuvant aromatase inhibitor therapy [8]. However, it should be noted that the effects of denosumab on BMD are relatively transient, as was observed in a study that evaluated the effects of discontinuing and restarting denosumab treatment in post-menopausal women with low-bone mass [9]. In this study, discontinuation of denosumab was associated with a BMD decrease of 6.6% at the lumbar spine and 5.3% at the total hip within the first 12 months after stopping treatment. This was paralleled by an increase in bone turnover marker levels as early as six months after discontinuation of denosumab treatment at dose levels comparable with those used in the early BC setting. These rates of bone loss were higher than those observed in placebo-treated patients at any point during the study. Although BMD benefits were restored by re-treatment, this rebound effect needs further consideration as this may be reflective of a bone microenvironment more conducive to tumour recurrence.

Thus, several large studies independently corroborate the benefit of bisphosphonates in the early BC setting in older or post-menopausal patients (low-estrogen environment) and support inclusion of ZOL as standard treatment for these patients. Further, these studies show that treatment with adjuvant bisphosphonates is safe and may provide sustained anticancer benefit—desirable treatment characteristics for this patient population with good prognosis and prone to recurrent disease.

## Advanced disease

The results of a randomized, controlled phase III trial comparing denosumab with ZOL in patients with advanced BC and at least one bone lesion demonstrated that denosumab significantly delayed the time to first on-study SRE (non-inferiority *P* < 0.001), the primary non-inferiority and secondary superiority endpoint of the study [5]. In addition, denosumab prolonged time to first and subsequent on-study SREs (*P* = 0.001). There were no differences in time to disease recurrence or overall survival. The data imply that the effects of either antiresorptive on the natural progression of malignancy are similar. However, survival differences may yet emerge with longer follow-up. Moreover, patients who went off study would likely have received bisphosphonates, a standard of care in this setting, and the effects of those and other off-study treatments on survival in this trial are unknown.

The effect of antiresorptives on cancer-related outcome may be influenced by both drug- and disease-related factors. First, nitrogen-containing bisphosphonates target a broad range of intracellular signal transduction intermediates, whereas denosumab acts exclusively by binding RANKL. Second, bisphosphonates have little or no systemic availability, unlike denosumab, so their effects are largely confined to bone. Conceivably, cancer-related outcomes may be influenced by effects on bone resorption alone, effects on tumour cells, or both. In addition, tumour type and disease burden may both be important determinants of treatment outcome. For example, in patients with multiple myeloma, wherein the majority of the cancer resides within the bone marrow, *post-hoc* analyses of phase III trial data in patients with malignant bone disease showed that, although patients with solid tumours had similar cancer-related outcomes, denosumab was associated with an increased risk of death versus ZOL in this subset of patients (HR = 2.26; 95% CI = 1.13–4.50; *n* = 180) [10, 11]. It is not currently known whether there may be a difference in treatment response based on number or size of bone lesions at baseline. However, exploratory analyses suggest that patients with bone metastases and elevated levels of osteolysis at baseline have improved survival with ZOL versus placebo [12, 13]. Moreover, control of skeletal disease may improve cancer-related outcomes, as suggested by a retrospective analysis showing that ZOL-mediated normalization of bone-turnover markers in patients with bone metastases from BC (*N* = 548) and elevated baseline osteolysis levels was associated with improved survival versus patients whose bone marker levels remained elevated (relative risk = 0.52; 95% CI = 0.34–0.78; *P* = 0.0017) [14].

The minimal inference from these data is that a reduction in bone turnover markers in patients with skeletal involvement is associated with improved outcomes, and that with the availability of new agents, clinicians may now consider sequencing of bone-conserving therapies. Recently, it was shown that denosumab may lower bone turnover markers in patients with elevated levels after previous bisphosphonate treatment [15]. However, it is unclear whether this denosumab-mediated normalization of bone markers correlates with improvements in disease outcomes. The role of markers in comparing treatment options therefore requires further study. Further trials are also needed to optimize sequencing strategies and dosing regimens as well as to identify baseline prognostic factors that may inform treatment decisions.

In addition to the effects in bone, ZOL may improve cancer-related outcome through extraskeletal effects. For example, recent exploratory data from the Z-ACT1 study show that ZOL induced rapid and sustained decrease in the proportion of metastatic BC patients (*N* = 29) with CTCs ≥5 (55% at baseline to 25% and 15% at study months 1 and 4, respectively) and, moreover, that patients with CTCs <5 had longer progression-free survival versus patients with CTCs ≥5 (296 versus 106 days; CTCs assessed at weeks 3–5) [16]. It should be noted that patient numbers from this study are small, and the effects of ZOL on cancer-related outcomes need to be confirmed in larger, prospective trials. However, these data are consistent with known prognostic significance of CTC level in metastatic BC [17, 18], and emerging evidence showing that ZOL decreases median CTC basal value from 20 (range, 0–981) to 10 cells (range, 0–362; P = 0.009) in BC patients with bone metastases [19]. These effects on CTCs are analogous to previously documented suppressive effects of ZOL on disseminated tumour cells [20]. These data warrant further prospective study of the anticancer benefit of bisphosphonates in metastatic BC patients prospectively stratified by baseline CTC status.

## Conclusions

With the availability of several antiresorptive agents, it is conceivable that skeletal- and cancer-related benefits may be considered collectively during treatment decisions throughout the disease continuum. Furthermore, the overall benefit may be optimized using patient-selection strategies based on menopausal status, age, CTCs, and/or bone metabolism markers. In addition to long-term risk–benefit considerations relating to bone health, the potential of antiresorptives to affect cancer-related outcomes may become an important consideration for treatment choice. Recent clinical evidence showing the potential anticancer activity of bisphosphonates, especially ZOL, suggests that re-evaluation of the benefits and roles of antiresorptive therapies is warranted.

## Figures and Tables

**Table 1:** Controlled studies of antiresorptive agents in breast cancer.

Study	Study description	Key results
Early/intermediate disease		
Coleman et al (AZURE) [1]	Placebo-controlled phase III study evaluating the benefit of ZOL in patients with early stage BC	No OS differences in overall population; however, subset analysis in patients showed that: Among post-menopausal patients, the 5-year rate of invasive DFS was 78.2% in the ZOL group and 71.0% in the control group (HR = 0.75; *P* = 0.02) Among patients who had undergone menopause >5 years before study entry, the 5-year OS rate was 84.6% in the ZOL group versus 78.7% in the control group (HR = 0.74; *P* = 0.04)
Gnant et al (ABCSG-12) [2]	Placebo-controlled phase III study evaluating the benefit of ZOL in pre-menopausal patients with early stage BC	DFS benefits observed at 48-month follow-up (HR = 0.74; log-rank *P* = 0.01) were maintained at 84 months (HR = 0.71; log-rank *P* = 0.011). Subset analyses at the 84-month follow-up show that DFS benefits appear to be driven by patients >40 years of age
Paterson et al (NSABP–B34) [3]	Placebo-controlled phase III study evaluating the benefit of oral clodronate in pre- and post-menopausal patients with non-metastatic BC stratified by HR and nodal status, and by age <50 or ≥50 years	In patients ≥50 years of age, clodronate improved: RFI: HR = 0.76; *P* = 0.05 BMFI: HR = 0.61; *P* = 0.024 nBMFI: HR = 0.63; *P* = 0.015 OS: HR = 0.80; *P* = 0.1
Mobus et al (GAIN) [4]	Randomized controlled, 2 × 2, factorial design trial of epirubicin, paclitaxel, and cyclophosphamide versus epirubicin, cyclophosphamide, paclitaxel, and capecitabine, each followed by either daily ibandronate or observation in patients with newly diagnosed, node-positive BC	No difference in the 3-year DFS (HR = 0.945; *P* = 0.59) or the 3-year OS (HR = 1.04; *P* = 0.80) between ibandronate versus observation in the ITT population
Advanced disease		
Stopeck et al (Amgen 136) [5]	Randomized phase III study comparing denosumab versus ZOL in patients with bone mets from advanced BC	Denosumab was superior to ZOL in delaying time to first on-study SRE (HR = 0.82; *P* = 0.01 superiority) and time to first and subsequent on-study SREs (rate ratio = 0.77; *P* = 0.001). No difference in survival or disease progression was noted

*BMFI* bone metastasis-free interval, *DFS* disease-free survival, *HR* hazard ratio, *ITT* intent to treat, *nBMFI* non-bone metastasis-free interval, *OS *overall survival,* RFI *recurrence-free interval,* SRE *skeletal-related event,* ZOL *zoledronic acid
